# Enamel Microhardness of Resin‐Infiltrated White Spot Lesions After Bleaching With and Without ACP: An In Vitro Study

**DOI:** 10.1155/ijod/9917511

**Published:** 2026-05-14

**Authors:** Pauravi Hegde, Shashi Rashmi Acharya, Arun Mayya, Kundabala Mala, Sandya Kini

**Affiliations:** ^1^ Department of Conservative Dentistry and Endodontics, DY Patil School of Dentistry, DY Patil (Deemed to be) University, Navi Mumbai, Maharashtra, India, dypatil.edu; ^2^ Department of Conservative Dentistry and Endodontics, Manipal College of Dental Sciences, Manipal Academy of Higher Education, Manipal 576104, Karnataka, India, manipal.edu; ^3^ Department of Conservative Dentistry and Endodontics, Manipal College of Dental Sciences, Mangalore, Manipal Academy of Higher Education, Manipal 576104, Karnataka, India, manipal.edu

**Keywords:** amorphous calcium phosphate, carbamide peroxide, dental bleaching, dental enamel, microhardness, remineralization, resin infiltration, tooth demineralization, Vickers hardness test, white spot lesions

## Abstract

**Background:**

White spot lesions (WSLs) represent the earliest clinical manifestations of enamel demineralization. Resin infiltration (RI) is a minimally invasive method used to mask WSLs for esthetic improvement; however, infiltrated enamel may remain prone to staining and surface degradation. Carbamide peroxide bleaching agents have been reported to reduce enamel microhardness, while the inclusion of remineralizing components such as amorphous calcium phosphate (ACP) may mitigate these effects.

**Objectives:**

To compare the microhardness of resin‐infiltrated enamel with artificial WSLs following the application of 22% carbamide peroxide bleaching systems with and without ACP.

**Methods:**

Thirty extracted noncarious maxillary premolars were prepared with artificial WSLs and treated with RI. The samples were randomly divided into three groups (*n* = 10): Group 1—RI only; Group 2—RI + 22% carbamide peroxide; and Group 3—RI + 22% carbamide peroxide + ACP. Vickers microhardness was measured at baseline (after RI and prior to intervention) and after 7 days. Data were analyzed using paired *t*‐tests for within‐group comparisons and one‐way ANOVA with Tukey’s post hoc test for intergroup comparisons.

**Results:**

One‐way ANOVA revealed a statistically significant difference in microhardness among the three groups (*p* = 0.039). The highest mean microhardness was observed in the group treated with 22% carbamide peroxide + ACP, demonstrating approximately a 10% increase in Vickers hardness compared with the resin‐infiltrated control group. Post hoc Tukey’s test showed a significant difference between the untreated and ACP‐treated groups (*p* = 0.031), while other comparisons were not statistically significant. Baseline microhardness values did not differ significantly among groups (*p* = 0.882), confirming comparability prior to intervention.

**Conclusion:**

Bleaching agents containing 22% carbamide peroxide, particularly when combined with ACP, were associated with higher posttreatment microhardness compared with the RI‐only group.

## 1. Introduction

White spot lesions (WSLs) are early clinical indicators of enamel subsurface demineralization [1, 2]. They arise from mineral loss within the interprismatic spaces, while the outer enamel layer may remain macroscopically intact [3]. Because these lesions are considered noncavitated and potentially reversible, minimally invasive treatment strategies are often favored in clinical care [4].

Resin infiltration (RI) is a microinterventional technique designed to occlude the porosities of demineralized enamel using low‐viscosity resin, improving optical masking of WSLs [5]. Although this procedure enhances short‐term esthetic outcomes, existing evidence indicates that infiltrated enamel may present altered surface behavior due to the hydrophilic resin’s capacity for fluid sorption and surface softening over time [6–8]. Unlike fluoride therapy, which acts through surface mineral dynamics, RI provides a primarily structural sealing effect and does not directly regenerate mineral content [9].

Carbamide peroxide bleaching agents are commonly used in home‐based tooth‐whitening protocols [10]. Experimental studies demonstrate that peroxide‐based bleaching can temporarily reduce enamel microhardness by affecting mineral‐density distribution and inducing surface changes [11–13]. Incorporation of remineralizing additives, particularly amorphous calcium phosphate (ACP), has been explored for its ability to deliver calcium and phosphate ions to the enamel surface. ACP is understood to act as a bioactive reservoir that may release mineral ions under aqueous conditions and contribute to hydroxyapatite deposition within surface porosities when conditions permit [12, 14, 15]. However, investigated effects vary across product formulations and study models, and the extent of hardness preservation following bleaching is not fully uniform in the literature. Limited evidence exists on the mechanical integrity of resin‐infiltrated enamel subjected to ACP‐modified bleaching. This study addresses this gap by evaluating the protective effect of ACP incorporated in 22% carbamide peroxide, a commonly prescribed, moderate‐strength home bleaching agent with established clinical safety.

From a public health and policy perspective, early interception of noncavitated carious lesions supports minimally invasive strategies aimed at preserving enamel structure and delaying restorative intervention. Evaluating the mechanical safety of esthetic procedures applied to early lesions remains relevant not only in ensuring that preventive and cosmetic approaches do not compromise enamel integrity but also in informing broader preventive oral health strategies.

This in vitro study aimed to compare the postbleaching enamel microhardness of resin‐infiltrated artificial WSLs following treatment with 22% carbamide peroxide bleaching systems with and without ACP, and to assess whether ACP addition is associated with a difference in microhardness recovery or retention.

## 2. Materials and Methods

The study was conducted at the Department of Conservative Dentistry and Endodontics, Manipal College of Dental Sciences, Manipal. Ethical clearance was obtained from the Institutional Ethics Committee (Approval Number 747/2018). Extracted human premolars used in this study were obtained from patients undergoing orthodontic treatment, with prior informed consent for their use in research. All procedures were carried out in accordance with the principles of the Declaration of Helsinki and institutional guidelines for the use of human biological materials.

### 2.1. Sample Size Calculation

The required sample size was estimated using 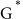
Power software Version 3.1.9.7. The sample size was estimated to apply one‐way ANOVA to compare the mean hardness between the three groups. A minimum of 10 participants per group (total *n* = 30) was required to detect an effect size of Cohen’s *f* = 0.6 at *α* = 0.05 and power = 0.80. An effect size (Cohen’s *f* = 0.6) was selected based on conventional benchmarks for large effects described by Cohen [16] and the magnitude of differences reported in previous in vitro studies evaluating enamel microhardness following bleaching and remineralization interventions [11–14].

### 2.2. Specimen Selection

Thirty extracted human maxillary premolar teeth with intact enamel surfaces and no clinical evidence of caries were included. Teeth exhibiting cracks, structural defects, restorations, or prior endodontic treatment were excluded. Soft tissue residues were removed using Gracey periodontal curettes (Hu‐Friedy Mfg. Co., Chicago, IL, USA). Teeth were disinfected in 5.25% sodium hypochlorite solution (Nice Chemicals Pvt. Ltd., Kochi, Kerala, India), rinsed with deionized water, and stored in distilled water for 20 h at room temperature before the experiment.

### 2.3. Specimen Preparation

Crowns were sectioned at the cementoenamel junction using a water‐cooled diamond precision saw (Isomet 1000, Buehler Ltd., Lake Bluff, IL, USA). The crown portions were embedded in transparent auto‐polymerizing acrylic resin blocks (DPI‐RR Cold Cure, Dental Products of India Ltd.). The buccal enamel surface was left exposed. Surface polishing was performed using silicon‐carbide abrasive paper discs (600‐, 800‐, 1200‐grit; Buehler Ltd., Lake Bluff, IL, USA) under water irrigation to standardize the test surface.

### 2.4. Artificial WSL Formation

A 4 mm × 4 mm window area was delineated on the buccal surface. The remaining areas were coated with transparent acid‐resistant nail varnish (Lakmé Absolute Gel Stylist, Hindustan Unilever Ltd., Mumbai, India). The exposed windows were immersed in 500 mL of demineralizing solution prepared according to the protocol described by White [17], consisting of acetic acid, calcium chloride dihydrate, potassium dihydrogen phosphate, potassium hydroxide, thymol, and methylhydroxydiphosphonate dissolved in distilled water (reagents: Merck KGaA, Darmstadt, Germany), maintained at pH 4.95 and 37 ± 0.2°C for 28 days to form standardized artificial, noncavitated WSLs. The pH was monitored daily using a digital pH meter (Oakton pH 700 Benchtop Meter, Cole‐Parmer India Pvt. Ltd., Mumbai, India) and adjusted when required using 10% hydrochloric acid or 10 M potassium hydroxide solutions (both analytical grade; Merck KGaA, Darmstadt, Germany).

### 2.5. RI of Artificial WSLs

RI was performed using commercially available kits according to manufacturer instructions. Lesions were conditioned with hydrochloric acid gel (Icon Etch, DMG Chemisch‐Pharmazeutische Fabrik GmbH, Hamburg, Germany) for 2 min, rinsed with deionized water, and air‐dried. Ethanol desiccation was done using 99% ethanol (Icon Dry, DMG Chemisch‐Pharmazeutische Fabrik GmbH, Hamburg, Germany). A low‐viscosity resin infiltrant (Icon Infiltrant, DMG Chemisch‐Pharmazeutische Fabrik GmbH, Hamburg, Germany) was applied to the window for 3 min and light‐cured for 40 s using an LED curing unit (Bluephase G2, Ivoclar Vivadent AG, Schaan, Liechtenstein). The resin was reapplied for 1 min and light‐cured again.

### 2.6. Bleaching Intervention

Specimens (*n* = 30) were randomized into three groups (*n* = 10 each):

• Group 1 (control): Resin‐infiltrated WSLs without bleaching.
• Group 2: Treated with home‐bleaching gel containing 22% carbamide peroxide (Whiteness Perfect, FGM Dental Products, Joinville, Santa Catarina, Brazil).
• Group 3: Treated with home‐bleaching gel containing 22% carbamide peroxide with ACP (NiteWhite ACP, Philips Oral Healthcare, Bothell, WA, USA).

Approximately 1 mm thickness of gel was applied on the enamel surface for 4 h daily for 7 consecutive days. Fresh bleaching gel was applied daily prior to each application cycle to ensure consistency of the active agent.

### 2.7. pH Cycling Regimen

Between bleaching applications, all samples underwent pH cycling to simulate dynamic ionic fluctuations of the oral environment. Specimens were stored in 500 mL of artificial saliva using a commercially available saliva substitute (Saliva Substitute, Roxane Laboratories, Columbus, OH, USA) at pH 7.0 and 37°C; the detailed ionic composition of the solution was not independently analyzed in this study. A daily acidic challenge consisted of 2 h of immersion in the same demineralizing solution, followed by 22 h of storage in artificial saliva. Samples were rinsed with deionized water when transferring between solutions to avoid ion carry‐over. The same pH‐cycling protocol was applied to all groups, including the control group, to ensure standardized posttreatment conditions.

### 2.8. Microhardness Testing

Microhardness was measured on the 4 mm × 4 mm resin‐infiltrated lesion window using a micro‐Vickers hardness tester (Matsuzawa MMT‐X7A, Matsusawa Co., Ltd.). The microhardness tester was calibrated according to the manufacturer’s instructions prior to measurements to ensure accuracy and consistency. A 100 g load was applied for 15 s, placing three indentations per specimen within the test window at ≥200 µm spacing to avoid interaction between adjacent indentations. All measurements were performed by a single operator on polished enamel surfaces to ensure consistency. The mean indentation Vickers hardness number (VHN) value per specimen was recorded. Baseline hardness was measured after RI and before bleaching, and final hardness was measured at 7 days postintervention.

### 2.9. Statistical Analysis

Data were entered in Microsoft Excel (Microsoft Corporation, Redmond, WA, USA) and analyzed using Jamovi (Version 2.3.24, The Jamovi Project, Sydney, Australia). Descriptive statistics (mean, SD, minimum, maximum) were computed. The baseline and postintervention VHNs were compared using a paired *t*‐test. One‐way ANOVA followed by Tukey’s post hoc test (*α* = 0.05) was used for intergroup comparison after verifying normality (Shapiro–Wilk test) and homogeneity of variance (Levene’s test). The level of significance was set at *p* < 0.05.

## 3. Results

Microhardness was evaluated using a Matsusawa Micro Vickers Hardness Tester and expressed in VHN. Table 1 summarizes the baseline groupwise microhardness (VHN). Tests of the underlying assumptions for ANOVA confirmed that the residuals were normally distributed (Shapiro–Wilk *W* = 0.970, *p* = 0.528) and that variances did not differ significantly across groups (Levene’s *F* [2, 27] = 0.021, *p* = 0.979). Baseline microhardness did not differ significantly among groups (ANOVA *F* [2, 27] = 0.126, *p* = 0.882), confirming comparability after randomization.

**Table 1:** Groupwise baseline microhardness (VHN).

Group	*n*	Mean^a^ (VHN)	SD	Min	Max
Resin infiltration (RI)	10	223.62	11.81	207.88	242.80
RI + 22% carbamide peroxide	10	221.72	12.97	201.40	247.30
RI + 22% carbamide peroxide + ACP	10	224.47	12.84	204.80	249.40

^a^ANOVA *F* (2, 27) = 0.126, *p* = 0.882, mean values did not differ significantly.

**Figure 1:**
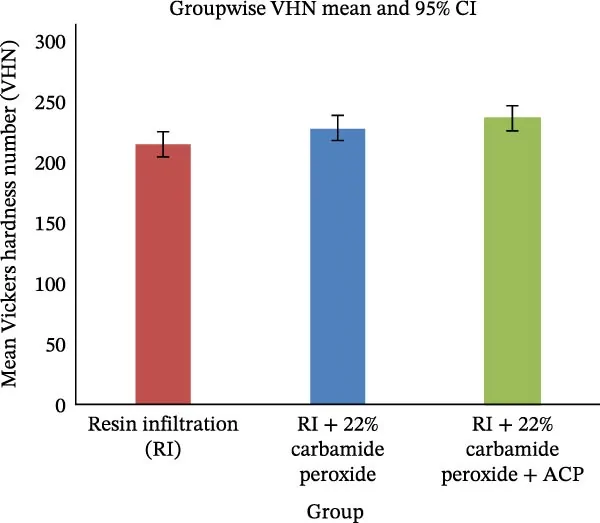
Groupwise comparison of postintervention Vickers microhardness (VHN) with 95% confidence intervals.

Table 2 summarizes the postintervention groupwise microhardness (VHN). Group 1 (RI) exhibited the least microhardness, and group 3 (RI + 22% carbamide peroxide + ACP) exhibited the highest microhardness (Figure 1).

**Table 2:** Groupwise postintervention microhardness (VHN).

Group	*n*	Mean^a^ (VHN)	SD	Min	Max
Resin infiltration (RI)	10	214.46	14.49	194.90	237.80
RI + 22% carbamide peroxide	10	227.22	17.71	202.80	258.10
RI + 22% carbamide peroxide + ACP	10	235.92	20.77	207.80	275.80

^a^ANOVA *F* (2, 27) = 3.661, *p* = 0.039; *η* ^2^ = 0.213, indicating statistically significant difference in mean values.

Tests of the underlying assumptions for ANOVA confirmed that the residuals were normally distributed (Shapiro–Wilk *W* = 0.969, *p* = 0.518) and that variances did not differ significantly across groups (Levene’s *F* [2, 27] = 0.722, *p* = 0.495). A one‐way ANOVA revealed a statistically significant difference in mean microhardness between the groups (*F* [2, 27] = 3.661, *p* = 0.039; *η* ^2^ = 0.213), indicating a large magnitude of effect based on conventional benchmarks adapted from Cohen [16] (0.01 = small, 0.06 = medium, and 0.14 = large).

Pre and postintervention comparison: The control group showed a significant reduction in VHN from baseline to postintervention (mean change [∆VHN] = −9.16, *p* = 0.004), while the carbamide peroxide group (∆VHN = + 5.50, *p* = 0.461) and the ACP group (∆VHN = + 11.45, *p* = 0.184) showed no statistically significant within‐group change.

### 3.1. Post Hoc Analysis (Postintervention Microhardness)

Tukey’s HSD test showed a significant difference between the RI group and the RI + 22% carbamide peroxide + ACP group (mean difference = −21.46, *p* = 0.031; Cohen’s *d* = 1.198). However, no significant differences were observed between the other pairs (Table 3). These comparisons demonstrated moderate effect sizes (Cohen’s *d* = 0.451–0.789), suggesting potentially meaningful differences despite the lack of statistical significance.

**Table 3:** Tukey’s post hoc test: pairwise comparison of postintervention microhardness (VHN).

Group comparison	Mean diff.	95% CI	*p*‐Value ^∗^	Cohen’s *d*
RI vs. RI + 22% carbamide peroxide	−12.76	−32.54 to 7.02	0.263	0.789
RI vs. RI + 22% carbamide peroxide + ACP	−21.46	−41.24 to −1.68	0.031 ^∗^	1.198
RI + 22% carbamide peroxide vs. RI + 22% carbamide peroxide + ACP	−8.70	−28.48 to 11.08	0.528	0.451

^∗^ *p*  < 0.05 is considered statistically significant.

## 4. Discussion

WSLs are recognized as the earliest clinical indicators of enamel demineralization, presenting as subsurface mineral loss within the interprismatic matrix while the external enamel surface remains intact [18]. Due to the heterogeneity of natural WSLs, artificial enamel‐window models are commonly used to standardize substrates for mechanical‐intervention comparisons.

RI has emerged as a preferred microinterventional strategy for the management of early enamel lesions due to its minimally invasive nature and strong optical masking potential [19]. In this study, RI was performed using the Icon system. The infiltrant fills and blocks subsurface micropores via capillary penetration but does not biologically redeposit calcium (Ca) or phosphate (P) ions, nor does it intrinsically regenerate hydroxyapatite crystallites [20]. Accordingly, postbleaching hardness change reflects residual enamel mineral behavior and ionic influences rather than resin‐intrinsic ion redeposition. Baseline microhardness values did not differ significantly among groups, confirming that the observed posttreatment differences are attributable to the experimental interventions rather than initial variability in enamel hardness.

Moderate‐to‐high‐concentration carbamide peroxide home bleaching agents remain among the most commonly prescribed esthetic interventions for stain reduction and enamel optical enhancement. In this study, bleaching was performed using 22% carbamide peroxide home‐bleaching gels (Whiteness Perfect and NiteWhite ACP), applied intermittently for 4 h/day for 7 days. A 4‐h daily application reflects a clinically simulated, intermittent contact regime that avoids unrealistic continuous exposure to peroxide, which has been associated in experimental studies with surface inorganic disruption, altered prism integrity, and temporary enamel softening [21–23]. This duration is considered moderate in experimental enamel research and is aligned with documented home‐bleaching contact periods used to study surface‐mechanics behavior [24, 25]. A 7‐day bleaching protocol was selected to simulate controlled short‐term exposure conditions commonly used in in vitro studies to evaluate early enamel surface changes while avoiding excessive alteration associated with prolonged exposure periods [22].

Microhardness was evaluated using a Vickers microhardness tester with a 100 g load for 15 s. The Vickers method is well‐validated for surface hardness evaluation of brittle, anisotropic dental enamel substrates [26–28]. While Knoop indentation shows higher orientation sensitivity, Vickers testing offers better diagonal accountability for controlled enamel surface evaluations [29–31].

In the present study, the group subjected to bleaching without ACP demonstrated higher mean microhardness values compared to the RI‐only group; however, this difference was not statistically significant. This may be related to the effects of the pH‐cycling regimen and the interaction of bleaching agents with the enamel surface. The alternating demineralization and remineralization phases, along with storage in artificial saliva, may facilitate ionic interactions at the enamel surface, contributing to changes in surface hardness [11]. Additionally, carbamide peroxide‐based bleaching agents have been reported to induce alterations in enamel surface characteristics, which may influence microhardness measurements under controlled in vitro conditions. However, within‐group comparisons did not demonstrate statistically significant changes in microhardness from baseline for either the carbamide peroxide or ACP‐containing groups. This indicates that the observed differences are reflective of relative intergroup variation rather than definitive increases in enamel surface hardness.

ACP dissociates into calcium‐ and phosphate‐rich ions in neutral or supersaturated media and may be associated with increased surface hardness through ionic interactions at the enamel surface [32–34]. In the present study, the 22‐h storage period in an artificial saliva reservoir after each bleaching cycle likely provided a neutral, ion‐supersaturated medium, which may facilitate the interaction of calcium and phosphate ions with the enamel surface. While hydroxyapatite nucleation prior to microindentation was not chemically or microscopically verified here, the highest VHN values observed in this group demonstrate a measurable association between ACP‐supplemented bleaching and increased residual enamel hardness in the infiltrated WSL window. This difference was statistically significant compared with the RI‐only group. This is consistent with previous studies reporting improved enamel surface hardness associated with calcium–phosphate‐containing bleaching systems. In a comparable in vitro investigation, Llena et al. [35] demonstrated that hydrogen peroxide and carbamide peroxide regimens produced similar enamel surface morphological shifts without measurable dentin hardening but that postbleaching short‐duration Ca/P‐with‐fluoride ion‐release Ca‐recovery activity was significantly increased only at the enamel‐surface interface and not within dentin tissues. Their results support the role of ionic calcium–phosphate reservoirs in contributing to increased enamel surface hardness following bleaching. Conversely, Almosa et al. [36] observed inferior hardness retention following RI alone, while calcium‐fluoride‐phosphate carriers showed more consistent enamel hardness regain. The artificial saliva used in this study was a commercially available saliva substitute intended to simulate oral conditions. However, its precise ionic composition, including calcium and phosphate concentrations, was not independently quantified or standardized. As the proposed mechanism of ACP relies on calcium and phosphate ion availability, the absence of direct ionic analysis represents a limitation. Therefore, the mechanistic interpretation should be considered inferential and based on established literature rather than direct experimental verification.

Notably, Icon infiltration does not uniformly compromise enamel surface hardness across different models. El‐Meligy et al. [37] reported an initial rise in enamel surface hardness following Icon infiltration but noted a rough, irregular enamel surface microtopography without evidence of true internal mineral regeneration .

The existing literature further indicates that mechanical outcomes from mineral‐enriched bleaching systems are formulation‐, exposure‐, and substrate‐dependent, rather than universally restorative. Nunes et al. [14] concluded that calcium‐based additives demonstrate a protective trend for enamel hardness after bleaching, but mechanical outcomes vary based on formulation, exposure parameters, and cycling model design. This selectively supports our findings by confirming that ionic Ca/P availability in added remineralizing complexes is likely to induce measurable mechanical surface‐hardness outcomes at the enamel‐prism interface where Ca/P may be associated with increased surface hardness despite structural pore occlusion effects from RI.

A simplified, 4 mm × 4 mm enamel‐window model cannot reproduce microbial biofilm dynamics, salivary enzyme‐mediated ion‐flux kinetics, pellicle maturation, or chromogen‐adhesion behavior, which are recognized determinants of surface staining and prism‐interface behavior. As such, although the controlled 7‐day window‐contact model allows surface‐specific sensitivity evaluation, interpretation of these findings should be limited to inorganic surface mechanics without assuming full biological lesion behavior.

Although a standardized demineralization protocol (White, 1987) was employed to produce artificial WSLs under controlled conditions, lesion depth and mineral loss were not directly quantified using cross‐sectional microhardness or imaging techniques. Therefore, uniformity of lesion severity was assumed based on the protocol rather than experimentally verified, which may limit reproducibility and comparability with other studies.

This in vitro study was designed to allow controlled evaluation of enamel microhardness; however, certain methodological considerations should be noted. The integrity of the surface layer following artificial lesion formation and acid etching was not directly evaluated. Therefore, the extent of surface erosion and the depth of resin penetration could not be confirmed, and the possibility that the infiltrant remained predominantly at the surface cannot be excluded. Although specimens were standardized and randomly allocated, intersample variability in enamel substrate cannot be completely excluded. In addition, the sample size was calculated based on an anticipated large effect size; however, the observed effect size in the present study was lower, suggesting that the study may have been underpowered to detect smaller intergroup differences. The microhardness was assessed at defined pre and posttreatment points; intermediate measurements during treatment were not performed, which may limit assessment of temporal changes in surface properties. Enamel prism orientation was not specifically standardized or analyzed, which may influence microhardness measurements due to the anisotropic nature of enamel. Although all measurements were performed by a single operator to reduce variability, intraoperator reliability was not formally assessed, and the exact spatial distribution of indentations within the test window was not mapped. These factors may introduce minor variability and should be considered when interpreting the results. Aging procedures such as thermocycling were not performed, and therefore long‐term thermal or mechanical effects on the resin–enamel interface were not assessed. In addition, surface roughness and color stability, which may influence clinical performance following bleaching, were beyond the scope of the present investigation. Microhardness testing was used as the primary outcome measure to evaluate mechanical surface changes; however, additional analyses such as surface roughness assessment, SEM or EDX evaluation, and evaluation of masking or bleaching efficacy were not included. Therefore, the findings represent a focused assessment of surface hardness rather than a comprehensive evaluation of all structural and esthetic outcomes. Future investigations incorporating aging protocols, surface characterization, and complementary analytical techniques may further clarify the long‐term behavior of resin‐infiltrated enamel subjected to ACP‐containing bleaching systems. In situ or clinical studies would help confirm the relevance of these findings under oral conditions.

## 5. Conclusion

RI provides structural pore sealing without direct mineral regeneration. Within the limitations of this in vitro model, bleaching with 22% carbamide peroxide, particularly when combined with ACP, was associated with higher posttreatment enamel microhardness compared with the RI‐only group. These findings suggest a potential influence of ACP on enamel surface hardness; however, further studies are required to confirm the underlying mechanisms and clinical relevance. Such evidence contributes to prevention‐oriented oral health strategies that emphasize early intervention and conservation of tooth structure.

## Author Contributions

**Pauravi Hegde**: conceptualization, methodology, investigation, data curation, writing – original draft. **Shashi Rashmi Acharya**: conceptualization, supervision, methodology, writing – review and editing, validation. **Arun Mayya**: formal analysis, data curation, writing – review and editing, visualization. **Kundabala Mala**: methodology, resources, writing – review and editing. **Sandya Kini**: formal analysis, validation, writing – review and editing.

## Funding

The authors have nothing to report.

## Conflicts of Interest

The authors declare no conflicts of interest.

## Data Availability

The data that support the findings of this study are available from the corresponding author upon reasonable request.
