# Correction to “Enamel Microhardness of Resin‐Infiltrated White Spot Lesions After Bleaching With and Without ACP: An In Vitro Study”

**DOI:** 10.1155/ijod/9842358

**Published:** 2026-09-01

P. Hegde, S. R. Acharya, A. Mayya, K. Mala, and S. Kini, “Enamel Microhardness of Resin‐Infiltrated White Spot Lesions After Bleaching With and Without ACP: An In Vitro Study,” *International Journal of Dentistry*, 2026 (2026): 9917511, https://doi.org/10.1155/ijod/9917511.

In the originally published article, the affiliation of Shashi Rashmi Acharya, Arun Mayya, Kundabala Mala, and Sandya Kini was incorrectly presented as a single affiliation, “Department of Conservative Dentistry and Endodontics, Manipal College of Dental Sciences Mangalore, Manipal Academy of Higher Education, Manipal, Karnataka, India,” associated with Manipal College of Dental Sciences, Mangalore.

The correct affiliations and author–affiliation assignments should read as follows:

**Shashi Rashmi Acharya, Arun Mayya, and Sandya Kini:**

Department of Conservative Dentistry and Endodontics, Manipal College of Dental Sciences, Manipal Academy of Higher Education, Manipal, Karnataka, India.

**Kundabala Mala:**

Department of Conservative Dentistry and Endodontics, Manipal College of Dental Sciences, Mangalore, Manipal Academy of Higher Education, Manipal, Karnataka, India.

This has been corrected in the published version of the paper.

We apologize for this error.

